# Genomic Analysis of a Hybrid Enteroaggregative Hemorrhagic *Escherichia coli* O181:H4 Strain Causing Colitis with Hemolytic-Uremic Syndrome

**DOI:** 10.3390/antibiotics11101416

**Published:** 2022-10-14

**Authors:** Angelina A. Kislichkina, Nikolay N. Kartsev, Yury P. Skryabin, Angelika A. Sizova, Maria E. Kanashenko, Marat G. Teymurazov, Ekaterina S. Kuzina, Alexander G. Bogun, Nadezhda K. Fursova, Edward A. Svetoch, Ivan A. Dyatlov

**Affiliations:** 1 Department of Culture Collection, State Research Center for Applied Microbiology and Biotechnology, Territory “Kvartal A”, 142279 Obolensk, Russia; 2 Department of Molecular Microbiology, State Research Center for Applied Microbiology and Biotechnology, Territory “Kvartal A”, 142279 Obolensk, Russia; 3 Department of Training and Improvement of Specialists, State Research Center for Applied Microbiology and Biotechnology, Territory “Kvartal A”, 142279 Obolensk, Russia

**Keywords:** *Escherichia coli*, hybrid pathotype, EHEC, EAHEC, O181

## Abstract

Hybrid diarrheagenic *E. coli* strains combining genetic markers belonging to different pathotypes have emerged worldwide and have been reported as a public health concern. The most well-known hybrid strain of enteroaggregative hemorrhagic *E. coli* is *E. coli* O104:H4 strain, which was an agent of a serious outbreak of acute gastroenteritis and hemolytic uremic syndrome (HUS) in Germany in 2011. A case of intestinal infection with HUS in St. Petersburg (Russian Federation) occurred in July 2018. *E. coli* strain SCPM-O-B-9427 was obtained from the rectal swab of the patient with HUS. It was determined as O181:H4-, *stx2-*, and *aggR*-positive and belonged to the phylogenetic group B2. The complete genome assembly of the strain SCPM-O-B-9427 contained one chromosome and five plasmids, including the plasmid coding an aggregative adherence fimbriae I. MLST analysis showed that the strain SCPM-O-B-9427 belonged to ST678, and like *E. coli* O104:H4 strains, 2011C-3493 caused the German outbreak in 2011, and 2009EL-2050 was isolated in the Republic of Georgia in 2009. Comparison of three strains showed almost the same structure of their chromosomes: the plasmids pAA and the *stx2a* phages are very similar, but they have distinct sets of the plasmids and some unique regions in the chromosomes.

## 1. Introduction

*Escherichia coli* is a bacterium that is widely distributed as a free-living in the environment and as an important member of the large intestine microbiota in humans and warm-blooded animals [1,2]. Although most *E. coli* are harmless commensals, some strains of this species are pathogenic and can induce diseases in humans and animals [3]. Human-pathogenic *E. coli* strains exhibit a wide spectrum of clinical manifestations, which are dependent on the virulence factors [4]. The *E. coli* genome is characterized by genetic mosaicism, high variability, and the ability to exchange genetic information [5]. In most cases, this exchange is carried out by the horizontal transfer of the bacterial mobile genetic elements such as plasmids, transposons, pathogenicity islands, and bacteriophages [6,7]. Pathogenic *E. coli*, according to their localization in the macro-organism and generated pathological processes, are divided into diarrheagenic (DEC) and extraintestinal (ExPEC) pathogens [8,9]. Seven pathotypes have been described for the DEC group, including enteropathogenic *E. coli* (EPEC), enterohaemorrhagic *E. coli* (EHEC), enterotoxigenic *E. coli* (ETEC), enteroinvasive *E. coli* (EIEC), enteroaggregative *E. coli* (EAEC), diffusely adherent *E. coli* (DAEC), and adherent-Invasive *E. coli* (AIEC), based on their virulence mechanisms, associated clinical symptoms, and consequences [10]. *E. coli* of different pathotypes contains the specific combinations of virulence genetic determinants located in the chromosome and plasmids [10,11].

Improvements in techniques allowing the better understanding of the genomic and virulence mechanisms among diarrheagenic *E. coli* led to detecting atypical hybrid *E. coli* strains combining genetic markers of different pathotypes [12,13,14]. Hybrid DEC strains carrying various combinations of virulence factors have emerged worldwide and have been reported as a public health concern [15]. The most well-known example is the *E. coli* O104:H4 strain, which caused a serious outbreak of acute gastroenteritis and hemolytic uremic syndrome (HUS) in Germany in 2011 [16]. This strain produced a Shiga toxin 2 (Stx2), a signature feature of the EHEC pathotype, and additionally carried a plasmid containing the genes coding to the aggregative adherence fimbriae (AAF)-mediating aggregative adherence in EAEC [17]. This bacterium was considered as a hybrid novel genetic lineage: enteroaggregative hemorrhagic *E. coli* (EAHEC) [18]. Even earlier, several cases of HUS and bloody diarrhea occurred in the Republic of Georgia in 2009, which were caused by *stx2*-positive *E. coli* strains of O104:H4 [19]. Comparative genome analysis showed that the Georgian strains were the nearest neighbors to the agents of the outbreak in 2011; only several structural and nucleotide differences were detected in the *stx2* phage genomes, the *mer*/*tet* antibiotic resistance island, and in the prophage and plasmid profiles of the strains [20]. A case of intestinal infection with HUS happened in St. Petersburg (Russian Federation) in July 2018. The pathogenic *E. coli* strain was obtained from the rectal swab of a patient with HUS. The strain was identified as *E. coli* O181:H4-, *stx2-* and *aggR*-positive. This genetic profiling indicated that the studied *E. coli* belonged to EAEC and EHEC at the same time. It was positive for the *chuA* gene and for the fragment of TspE4.C2, which made it possible to assign them to the B2 phylogenetic group. In general, *E. coli* strains of the B2 phylogenetic group carry more virulence factors compared with the strains belonging to other phylogenetic groups (A, B1, D), which are associated with a more pronounced ability to colonize intestinal mucosa [21].

*E. coli* serogroup O181 is a relatively new; it was included in the serotyping scheme for *E. coli* in 2004 [22]. Sporadically, *E. coli* O181 were isolated from the patients with diarrhea, from meats and meat products, as well as from livestock wastewater and environmental objects [23,24,25,26]. The aim of this work is to characterize the genetic properties of the new hybrid enteroaggregative and Shiga-toxin-producing *E. coli* strain of O181:H4, which was isolated from the patient with HUS in Saint Petersburg, Russian Federation, in 2018.

## 2. Results

### 2.1. Bacterial Strain, Pathotype Identification, and Susceptibility to Antimicrobials

The *E. coli* clinical strain SCPM-O-B-9427 was isolated from a rectal swab of the patient with HUS in Saint Petersburg, Russian Federation, in 2018, and deposited in the State Collection of Pathogenic Microorganisms and Cell Cultures “SCPM-Obolensk”. Pathotype identification revealed that this strain belonged to both EHEC and EAEC (EAHEC), according to commercial assay AmpliSens^®^ Escherichioses-FRT and because it carried both *stx* and *aggR* genes, according to The European Union Reference Laboratory method. Multiplex PCR for phylogroup identification was positive for the *chuA* gene and for the fragment of TspE4.C2, which allows to assign the strain SCPM-O-B-9427 to the B2 phylogenetic group.

The strain SCPM-O-B-9427 was susceptible to amoxicillin/clavulanic acid, cefotaxime, ceftazidime, cefoperazone/sulbactam, cefepime, aztreonam, imipenem, meropenem, amikacin, gentamicin, netilmicin, fosfomycin, nitrofurantoin, and trimethoprim/sulfamethoxazole and resistant to ampicillin and ciprofloxacin (Table 1).

### 2.2. Genomic Characteristics of EAHEC Strain SCPM-O-B-9427

The complete genome assembly of the strain SCPM-O-B-9427 contained one chromosome and five plasmids, one of which is homologous with virulence plasmid pAA–pSCPM-O-B-9427-2, encoding an aggregative adherence fimbria I (AAF/I). The major genomic characteristics are summarized in Table 2 and are shown in comparison to two hybrid EAHEC strains of O104:H4: 2011C-3493, which caused large German outbreak in 2011, and 2009EL-2050, which caused the group outbreak in 2009 in Georgia. The chromosome sizes of the strains are comparable and slightly different, with identical GC content. The total numbers of genes, including RNA genes, are about the same amount, but the chromosome of the strain SCPM-O-B-9427 contained more pseudogenes.

### 2.3. Genotypic Profiling of the EAHEC Strain SCPM-O-B-9427

Genoserotyping of the strain SCPM-O-B-9427 was performed by extracting the sequences of the *wzx* gene (identity 100%, AB812078) and *wzy* gene (identity 99.92%, AB812078), which confirmed the affiliation of *E. coli* SCPM-O-B-9427 with the serogroup O181. The *fliC* gene was assigned to the type H4 (identity 100%, AJ605764). The *stxA* and *stxB* genes coding Shiga toxin Stx2a subunits A and B (identity 100%, LC645441), cluster genes *aggDCBA* coding the aggregative adhesion fimbriae (AAF), and the transcriptional activator *aggR* (identity 100%, CP003291) were detected in the genome.

In silico multi-locus sequence typing (MLST) based on seven loci of house-keeping genes by the Achtman’s MLST scheme database (*adk*_6, *fumC*_6, *gyrB*_5, *icd*_136, *mdh*_9, *purA*_7, *recA*_7) showed the strain SCPM-O-B-9427 belonging to ST678 [27]. The same sequence type was identified for the strain *E. coli* 2011C-3493 O104:H4 that caused the large German outbreak in 2011 and for the strain *E. coli* 2009EL-2050 O104:H4 that was isolated in the Republic of Georgia, in 2009.

Several chromosomal-located virulence genes were identified in the strain SCPM-O-B-9427 as well as in the genomes of two above-named EAHEC strains of O104:H4. The strain SCPM-O-B-9427 was positive for *aaiC* gene (coding type VI secretion protein), *capU* (hexosyltransferase homolog), *fyuA* (siderophore receptor), *gad* (glutamate decarboxylase), *iha* (adherence protein), *irp2* (high-molecular-weight protein 2 non-ribosomal peptide synthetase), *iucC* (aerobactin synthetase), *iutA* (ferric aerobactin receptor), *lpfA* (long polar fimbriae), *pic* (serine protease autotransporters of Enterobacteriaceae, SPATE), *sigA* (serine protease), and *terC* (tellurium ion resistance protein). However, the differences between three EAHEC strains were found: compared to the strains of O104:H4, the strain SCPM-O-B-9427 is negative for the *neuC* gene coding the polysialic acid capsule biosynthesis protein; and the strain 2011C-3493 is negative for the *bor* gene coding the serum resistance lipoprotein, while the strains SCPM-O-B-9427 and 2009EL-2050 are positive. In addition to the *aggDCBA* cluster and *aggR* regulator gene, other EAEC genetic determinants were detected in all three EAHEC strains: *sepA* gene (Shigella extracellular protein A), *aap* gene (dispersin), and *aat* operon (factor of adhesion). Other *E. coli* virulence genes such as *fimH*, *sfa*, *papA*, *hylA*, *cnfl*, *aer*, and *afaC* were not detected.

### 2.4. Chromosomal SNPs in the EAHEC Strains SCPM-O-B-9427, 2011C-3493, and 2009EL-2050

**Figure 1:**
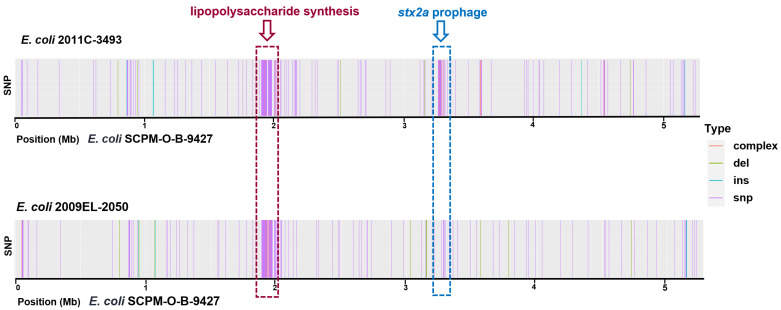
Distribution of the sequence variations at a genomic position. Chromosome mapping of EAHEC strains 2011C-3493 and 2009EL-2050 was performed against of the strain SCPM-O-B-9427 chromosome.

Chromosomes of the strains SCPM-O-B-9427, 2011C-3493, and 2009EL-2050 were compared using Snippy software for detailed genomic analysis. A comparison between the strains SCPM-O-B-9427 and 2011C-3493 revealed 1326 SNPs (959 synonymous, 242 non-synonymous, and 125 intergenic) and 26 insertions and deletions; 19 of them were intergenic. On the contrary, the difference between SCPM-O-B-9427 and 2009EL-2050 chromosomes was significantly less: 1053 SNPs were identified (793 synonymous, 180 non-synonymous, and 80 intergenic) as well as 20 insertions and deletions, and 14 of them were intergenic. Revealed SNPs were not evenly distributed along the chromosome but clustered in a few regions; the biggest one was the region coding lipopolysaccharide synthesis. The distribution of SNPs, insertions, deletions, and the chromosome complex of the strain SCPM-O-B-9427 compared to the strains 2011C-3493 and 2009EL-2050 is shown in Figure 1.

### 2.5. Chromosomal Structural Comparison of the Strains SCPM-O-B-9427, 2011C-3493, and 2009EL-2050

**Figure 2:**
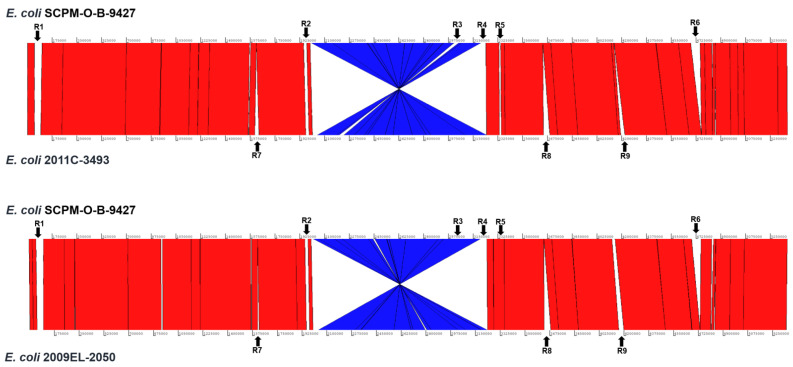
The Artemis comparison of the strain SCPM-O-B-9427 chromosome with chromosomes of the strains 2009EL-2050 and 2011C-3493. Red vertical lines correspond to the regions that are highly homologous and are oriented in the same order, and blue vertical lines depict a region whose orientation is reversed between strains. Arrows on the top and bottom indicate white lines showing that unique regions are non-homologous or are absent in another strain.

We studied the chromosomal architectures of the hybrid EAHEC strains SCPM-O-B-9427, 2009EL-2050, and 2011C-3493, which are very similar in overall structure, without rearrangements detected using Artemis (Figure 2). However, significant differences between genomes were revealed consisting of one extended inverted region in the genome of the strain SCPM-O-B-9427 and unique regions for each of three EAHEC strains. The features of non-homologous unique regions of the strains SCPM-O-B-9427, 2009EL-2050, and 2011C-3493 are listed in Table 3. The region R1 of the strain SCPM-O-B-9427 is not homologous to the same regions of the strains 2011C-3493 and 2009EL-2050. Since the strains belong to different serogroups, the region R2 carries genes are involved in lipopolysaccharide synthesis (O104 and O181 antigen gene clusters).

The strains 2011C-3493 and 2009EL-2050 are very similar genetically, and the regions of diversity with the strain SCPM-O-B-9427 are partly matched. Three unique regions (R7, 27.8 kbp; R8, 44.6 kbp; and R9, 30.2 kbp) of the strain 2011C-3493 are absent in the strain SCPM-O-B-9427; two of them were related to prophages; these prophages are truncated in the genome of the strain 2009EL-2050. Interestingly, the strains SCPM-O-B-9427 and 2009EL-2050 have a prophage (R3), which is missed in the strain 2011C-3493. The region R6 of the strain SCPM-O-B-9427 is unique and carrying the genes coding the type II secretion system; the region R4 of this strain is not homologous to the same regions of the strains 2011C-3493 and 2009EL-2050. The *stx2a* prophage (R5) is almost identical in the strains SCPM-O-B-9427 and 2009EL-2050, and there is a large number of SNPs in some genes in the strain 2011C-3493 (Table 3).

### 2.6. Comparison of the stx2a Prophages Located in the Genomes of the Strains SCPM-O-B-9427, 2011C-3493, and 2009EL-2050

**Figure 3:**
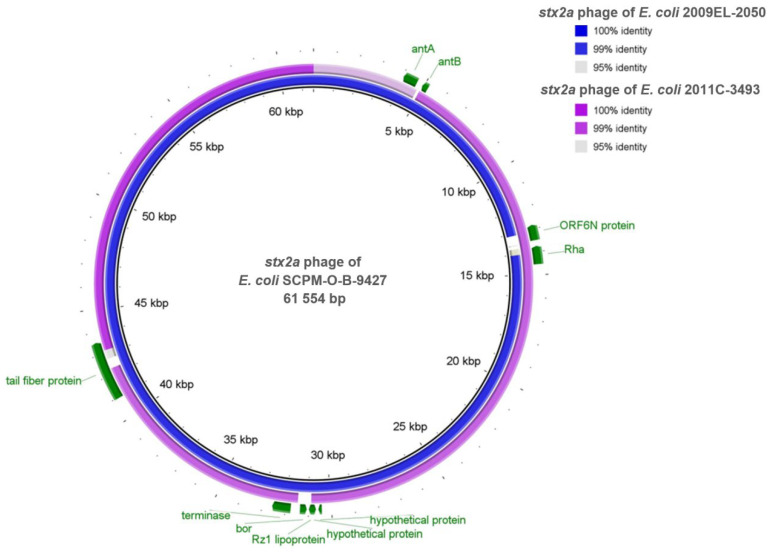
BRIG diagram of *stx2a* phages comparison. From center to outside: SCPM-O-B-9427 (black ring), 2009EL-2050 (blue ring), and 2011C-3493 (purple ring). The genes of the strain SCPM-O-B-9427 (colored green) show the regions of differences with the strains 2009EL-2050 and 2011C-3493.

We performed a comparison of the *stx2a*-carrying prophages of the strains SCPM-O-B-9427, 2011C-3493, and 2009EL-2050 using BRIG (Figure 3). The comparative analysis showed that the *stx2a* phage of the strain SCPM-O-B-9427 is highly homologous to the phage of the strain 2009EL-2050 and distinguished from that of the strain 2011C-3493. The strains SCPM-O-B-9427 and 2009EL-2050 carried a *bor* gene, which may be involved in serum complement resistance, but the strain 2011C-3493 does not have this gene. The second difference between the strain 2011C-3493 and other two strains is in sequence of putative tail fiber protein, which is 133 amino acids shorter. The third site of difference is a deletion, which perturbed homologous antirepressor proteins genes (*antA* and *antB*); the anti-termination protein gene is absent in the strain 2009EL-2050.

Nucleotide sequence alignment of the *stx2a*-carrying phages of three EAHEC strains was carried out by Snippy. A comparison between prophages of SCPM-O-B-9427 and 2011C-3493 strains showed 198 SNPs (132 synonymous, 43 non-synonymous, and 23 intergenic) and 6 insertions and deletions, where 3 of them are intergenic. The changes of nucleotides did not affect the *stx* genes, which were the most SNPs detected in the genes coding hypothetical proteins and putative endolysin. The number of SNPs between the *stx2a* phages of the strains SCPM-O-B-9427 and 2009EL-2050 is only five. One of them is intergenic, one is a non-synonymous change in each of two genes (hypothetical protein, putative tail fiber protein), and one is a synonymous change in each of two genes (repressor protein CI, Shiga toxin 2 subunit A).

### 2.7. The Plasmids of the Strain SCPM-O-B-9427

**Figure 4:**
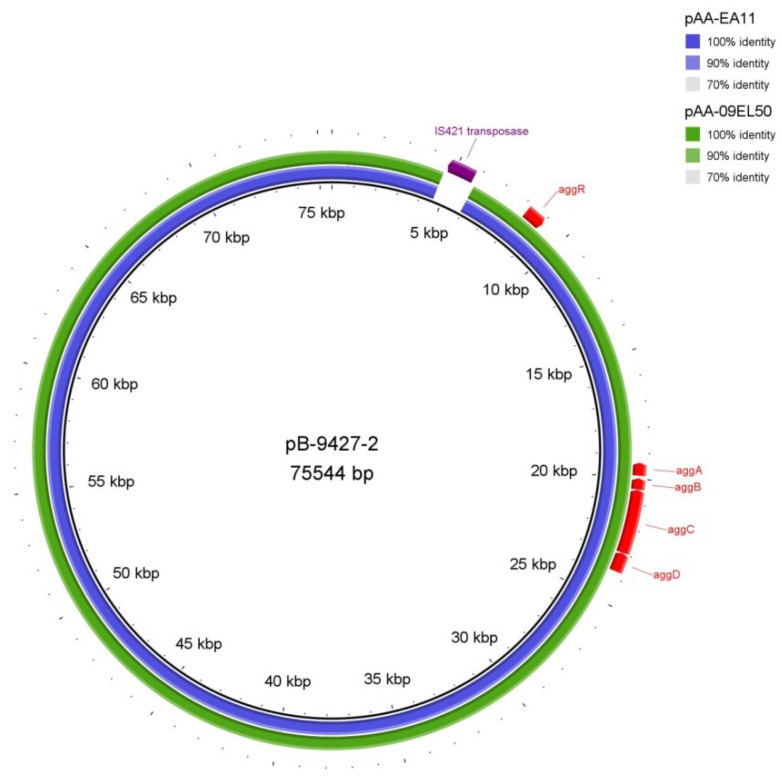
BRIG diagram of pAA plasmids comparison. From the center to outside: pB-9427-2 (black ring), pAA-09EL50 (blue ring), and pAA-EA11 (green ring). The cluster genes *aggDCBA* coding aggregative adhesion fimbriae (AAF) and transcriptional activator aggR are indicated (colored red). An additional insertion caused by IS4-like element IS421 family transposase in pB-9427-2 is shown (colored violet).

Five plasmids were identified in the strain SCPM-O-B-9427. Plasmid sequences were compared using BLASTn to the NCBI nucleotide database to identify their closest matches, which are given in Table 4. The plasmid pB-9427-2 (CP086261) is homologous to pAA plasmid, which harbored several EAEC-specific virulence loci, including *aggDCBA* cluster, *aggR* gene, *aatPABCD* operon, *sepA* gene, and *aap* gene. Importance of pAA in disease severity has been demonstrated: it was involved in the host–pathogen interaction [28] (Berger et al., 2016). We compared the plasmid pB-9427-2 with homologous plasmids pAA-EA11 and pAA-09EL50 of the strains 2011C-3493 and 2009EL-2050. The plasmids do not have rearrangements; the size of pB-9427-2 was 1331 bp bigger due to an additional insertion caused by IS4-like element IS421 family transposase (Figure 4).

The nucleotide sequence comparison of pB-9427-2 with 2011C-3493_pAA-EA11 by Snippy showed 39 SNPs (19 synonymous, 11 non-synonymous, and 9 intergenic) and 5 insertions; and with 2009EL-2050_pAA-09EL50, it showed 35 SNPs (21 synonymous, 10 non-synonymous, and 4 intergenic) and 7 deletions (four intergenic) and 1 insertion in *finO* (conjugal transfer fertility inhibition protein). The changes of nucleotides did not affect the virulence genes, and the most SNPs were detected in *umuC* (DNA polymerase V subunit), *finO*, *traX* (conjugal transfer pilus acetylation protein), *traI* (conjugal transfer nickase/helicase), and in transposase (IS110 family protein).

Another four replicons of the strain SCPM-O-B-9427 were not homologous to the plasmids of the strains 2011C-3493 and 2009EL-2050 (pESBL-EA11, pG, and p09EL50) (Table 2). The plasmids of the strain SCPM-O-B-9427 did not carry any antibiotic resistance genes, and there were not identified any significant virulence factors on the plasmid pB-9427-5 except *celb* gene (endonuclease colicin E2).

### 2.8. Phylogenetic Analysis for E. coli O181 and O104:H4

**Figure 5:**
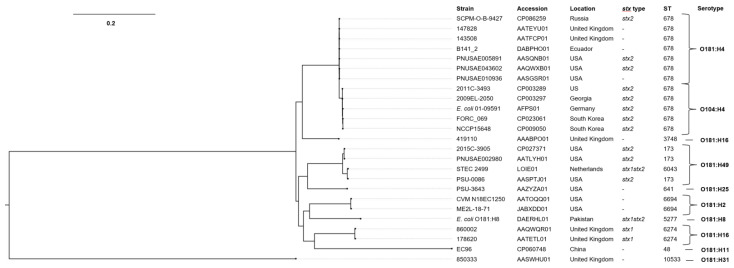
Neighbor-joining phylogenetic tree based on core SNPs, showing the phylogenetic relationship between chromosomes of EAHEC strain SCPM-O-B-9427 and *E. coli* O104:H4 and *E. coli* O181 strains. The scale bar shows the expected number of substitutions per site. Bar, 0.2 substitutions per nucleotide position.

To clarify the relationship of the hybrid EAHEC strain SCPM-O-B-9427 and several *E. coli* strains of O104:H4 and O181 found in the database NCBI, a phylogenetic tree based on the core chromosomal SNPs using Wombac was built. The tree was represented on 96,244 SNPs of 25 strains (Figure 5). The *E. coli* strains were grouped due to their sero- and MLST sequence type. The hybrid strain SCPM-O-B-9427 was very closely related to the group of O181:H4 and O104:H4 strains while not to the strains of O181 non-H4 serogroups.

**Figure 6:**
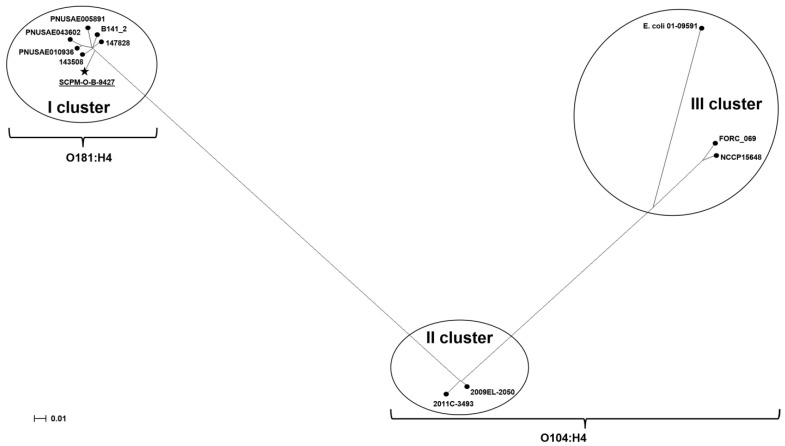
Neighbor-joining phylogenetic tree based on core SNPs, showing the phylogenetic relationship between chromosomes of EAHEC strain SCPM-O-B-9427 and *E. coli* O104:H4 and *E. coli* O181:H4 strains. The scale bar shows the expected number of substitutions per site. Bar, 0.01 substitutions per nucleotide position.

For a better understanding of the relationship within the group of the strains belonging to ST678, a second phylogenetic tree based on 2115 core SNPs of 12 strains was built. *E. coli* strains formed three clusters: the first one included the strains of O181:H4 serotype; the second included the strains of O104:H4 serotype (caused outbreaks in Germany, 2011 and Georgia, 2009) and the third the other O104:H4 strains (Figure 6). The strains from the second and the third clades are hybrid, carrying both the *stx2* gene and *aggDCBA* cluster. The difference between the second and the third clades was the carriage of fimbriae AAF/I or fimbriae AAF/III gene cluster, respectively.

On the phylogenetic tree, the strains of O181:H4 are spaced from the center of the clade and do not form a well-defined subclade. The performed comparison of the SCPM-O-B-9427 chromosome with other *E. coli* O181:H4 genomes showed that the core SNP numbers varied from 133 to 159. Major strains carried genes coding fimbriae AAF/I; only three of them including the strain SCPM-O-B-9427 additionally carried the *stx2* genes and were attributed as EAHEC.

We compared the sequences of *stxA* and *stxB* genes of O181:H4 strains (Figure 6), which were attributed to the 2a type. Significant differences were revealed between named genes of the strain SCPM-O-B-9427 and other two strains of this clade, PNUSAE005891 and PNUSAE043602. The strains PNUSAE005891 and PNUSAE043602 have identical sequence of *stx2* genes. Although StxA amino acid sequences are the same for the three strains, i.e., PNUSAE005891, PNUSAE043602, and SCPM-O-B-9427, seven synonymous SNPs were identified in the *stxA* gene of the strain SCPM-O-B-9427. Moreover, two synonymous SNPs and one SNP that led to a change from alanine to valine were detected in the *stxB* gene of the strain SCPM-O-B-9427 compared to the same gene of the strains PNUSAE005891 and PNUSAE043602.

We also compared the *stx2a*-carrying phage sequence of the strain SCPM-O-B-9427 with the same phage sequences of the strains PNUSAE005891 and PNUSAE043602. In NCBI Genome, the results of whole-genome sequencing of these strains are the assemblies of contigs. In the strain PNUSAE005891, the *stxA* and *stxB* genes were located in the contig 51 (AASQNB010000051, 21,013 bp) and in the strain PNUSAE043602 in the contig 30 (AAQWXB010000030, 73,345 bp). Sequence alignment of the *stx2a* phage of the strain SCPM-O-B-9427 and the contig 30 of the strain PNUSAE043602 showed only 11% query cover (the region of *stx* genes) and 96.24% identity of this region. It was pointed out that the *stx2a*-carrying phages have a different genetic structure. BLAST search revealed the closest to the prophage of the strain PNUSAE043602 sequence of the *stx2a*-carrying phage Stx2_12E129_yecE DNA (LC567842, 46,100 bp) with 82% query cover and 94.64% identity. Comparison of the contig 30 of the strain PNUSAE043602 and assembly PNUSAE005891 revealed that the *stx2a*-carrying phage of the second strain fell to pieces, but apparently, they are the similar.

## 3. Discussion

The conception about several well-classified pathotypes of DEC *E. coli* based on the presence of specific virulence factors directly related to disease development existed before the German outbreak in 2011. This conviction collapsed due to the fact that this severe outbreak was caused by a hybrid strain bearing the virulence factors of enteroaggregative *E. coli* and Shiga-toxin-producing *E. coli* simultaneously [16,17]. After that, many studies have shown that *E. coli* strains combining genetic markers belonging to different pathotypes are more frequent than previously thought [12,13,14,15]. Hybrid DEC are associated with more serious diseases and may frequently progress to HUS, which could be explained by producing a set of proteins involved in intestinal colonization, leading to persistent diarrhea and facilitating Stx absorption [29].

We present the description of a new hybrid EAHEC O181:H4 strain obtained from the rectal swab of a HUS patient in St. Petersburg (Russian Federation) in July 2018. The strain was investigated by whole-genome sequence analysis in this study.

The genoserotyping in silico confirmed that the strain SCPM-O-B-9427 was attributed to O181:H4 serogroup and to the sequence type ST678. The same ST was identified for the strains caused the large German outbreak in 2011 and the bloody diarrhea outbreak in the Republic of Georgia in 2009, but these strains belonged to O104 serogroup [20]. Affiliation to the same ST and similar sets of virulence genes suggested a close genetic relationship between these strains belonging to different serogroups. We found six assemblies of *E. coli* O181:H4 draft genomes in the NCBI database without detailed information about isolation sources and hosts. All of them belonged to ST678 and carried the *aggR* gene, and only two possessed *stxA* and *stxB* genes. Considering these data, it was not surprising that the phylogenetic relationship of *E. coli* O181:H4 strains based on core SNPs were very closely related to *E. coli* O104:H4.

Comparison of the strain *E. coli* SCPM-O-B-9427 with *E. coli* strains 2011C-3493 and 2009EL-2050 showed almost the same structure of their chromosomes and high similarity of two pAA-carrying plasmids the major virulence factors and *stx2a*-bearing prophages. There were identified few SNPs in the homologous parts of the chromosomes. However, at the same time, they were variable from each other and have unique differences. Hence, these strains carried distinct sets of the plasmids and unique regions in the chromosomes. Thus, the compared strains are close relatives; probably, they have the same ancestor, but different genetic events occurred during their evolution.

Unfortunately, study of the EAHEC strain SCPM-O-B-9427 genetic characteristics does not allow answering the question about the origin and evolution of this pathogen. We can suppose the hypothesis about the genesis of the EAHEC strain O104:H4 that caused severe foodborne infection in Germany in 2011 [16]. The proposed development model of this strain derivation consists of the acquisition of a *stx2a*-converting prophage into an EAEC strain by transduction. The Stx-converting phages were considered as highly mobile genetic elements capable of infecting a susceptible bacterium and leading to positive Stx-conversion. However, apart from the transduction of the *stx2a* phage in an EAEC cell, it should be introduced into the chromosome in appropriate location, be properly functioned, and be stably inherited. In some cases, the infection of DEC strains by Stx-converting phages has been carried out in experiments. The emergence of hybrid EAHEC strains is not an often-occurring event, which can be explained by different susceptibility of EAEC strains to the Stx-converting phages. Probably, the EAEC of O104:H4 and O181:H4 were competent recipients of Stx-converting phages; moreover, they are very genetically close to each other. It was revealed in our study that the *stx2a* prophages of the strains SCPM-O-B-9427 and 2009EL-2050 are almost identical, while they were somewhat different from the *stx2a* prophage of the strain 2011C-3493; nevertheless, all three *stx2a* prophages are very similar. Interestingly, the *E. coli* strains PNUSAE005891 and PNUSAE043602 of the O181:H4 serotype carried different *stx2a* prophages compared to that of the strain SCPM-O-B-9427. This fact may indicate an increased sensitivity of *E. coli* of different origins to the Stx-phage infection. The forming of new hybrid DEC strains has high threat potential to humans due to the combination of the damages induced by Stx on susceptible cells and the aggregative, adherence-mediating fimbriae allowed to colonize the gastrointestinal tract, which can lead to disease worsening and the development of HUS.

*E. coli* are characterized by a high degree of genetic heterogeneity, high genome plasticity, and the ability to exchange genetic information and thereby increase virulence, to acquire drug resistance, and eventually improve the adaptation to different environments, keeping *E. coli* as a successful bacterium in various ecological niches.

Although the strain SCPM-O-B-9427 and the “Georgian strain” caused not-so-large foodborne outbreaks compared to that of the “Germany strain”, some patients developed HUS. The emergence of O181:H4 EAHEC strain, phylogenetically related to the Shiga-toxin-producing *E. coli* of O104:H4, show that new genetic variants continuously formed in this bacterial species. Therefore, genetic research is essential for detecting and controlling the spread of new variants of pathogenic bacteria.

## 4. Materials and Methods

### 4.1. Bacterial Strains Used in This Study

*Escherichia coli* strain SCPM-O-B-9427 was obtained from the patient’s rectal swab received from The Center of Hygiene and Epidemiology in Saint Petersburg and deposited into The State Collection of Pathogenic Microbes of The State Research Center for Applied Microbiology and Biotechnology, Obolensk. It was not needed to obtain permission from the Ethics Committee to conduct the study. The name of the strain does not contain personal data about the patient.

### 4.2. Strain Isolation, Growing, Identification, and Susceptibility Testing

A clinical case of intestinal infection was registered in Saint Petersburg, Russian Federation, in July 2018. Bacterial isolates were collected according to the Methodical Recommendations MR 4.2.2963-11 “Methods for laboratory diagnostic of the infections caused by Shiga-toxin-producing *E. coli* (STEC-cultures) and detection of STEC pathogens in food”. *E. coli* isolates were grown on the nutrient media “Enrichment media for Enterobacteriaceae number 3” (SRCAMB, Obolensk, Russia) and “MacConkey agar” (HiMedia, Mumbai, Maharashtra, India) at 37 °C for 18 h. Bacterial species identification was completed using API-20 biochemical test system (Biomereux, Marcy-l’Étoile, Auvergne-Rhône-Alpes, France) and Vitek 2 Compact instrument (Biomereux, Marcy-l’Étoile, Auvergne-Rhône-Alpes, France) using VITEK 2 GN ID card (Biomereux, Marcy-l’Étoile, Auvergne-Rhône-Alpes, France). Bacterial isolates were stored in 10% glycerol at minus 70 °C.

Antimicrobial susceptibility testing was performed on Vitek2 Compact system (BioMérieux, Marcy-l’Étoile, Auvergne-Rhône-Alpes, France) using AST-N101 card (BioMérieux, Marcy-l’Étoile, Auvergne-Rhône-Alpes, France). The interpretation was made using requirements of the “The European Committee on Antimicrobial Susceptibility Testing (EUCAST), version 12.0, 2022 (http://www.eucast.org) (access on 5 September 2022). *Escherichia coli* ATCC 25922 strain was used as quality control.

### 4.3. DNA Isolation and Pathotype and Phylogroup Identification

DNA extraction was performed using a nucleic acid extraction kit AmpliSens^®^ RIBO-prep (InterLabService, Moscow, Russia). Pathotype detection was performed using commercial assay AmpliSens^®^ Escherichioses- FRT (InterLabService, Moscow, Russia). Detection of *stx* and *aggR* genes were completed according to The European Union Reference Laboratory method (EU Reference Laboratory VTEC, Rome, Italy, https://www.iss.it/about-eu-rl-vte) (access on 9 September 2021). Multiplex PCR for phylogroup identification was performed according to Clermont et al., 2013 [30].

### 4.4. Whole Genome Sequencing, Assembly and Annotation

DNA isolation was performed by CTAB method [31]. WGS was carried out using Nextera DNA Library Preparation Kit (Illumina, San Diego, CA, USA) and MiSeq Reagent Kits v3 (Illumina, San Diego, CA, USA) for platform Illumina MiSeq (Illumina, San Diego, CA, USA). Long reads were obtained using Rapid Barcoding Kit RBK004 and flowcell R9.4.1 on MinION platform (Oxford Nanopore, Oxford Science Park, GB). WGS was performed by running MinKNOW software v. 21.06.13 (Oxford Nanopore, Oxford Science Park, GB); basecalling was performed with Guppy v. 5.0.16 (Oxford Nanopore, Oxford Science Park, GB) with defaults parameters [32]. Short and long raw reads were used to obtain the hybrid assembly of the strain using Unicycler v. 0.4.7 software (The University of Melbourne, Victoria, Australia) with default settings that included primary filtering and quality control [33]. Annotation was carried out by NCBI Prokaryotic Genome Annotation Pipeline (PGAP) v. 5.3 (National Center for Biotechnology Information, Bethesda, MD, USA) [34].

### 4.5. Whole Genome Analysis

Serotyping and definition of virulence and resistance genes of complete genome was performed in silico using online resources SerotypeFinder 2.0 [35], VirulenceFinder 2.0 [36], ResFinder 2.0 [37] of the Center for Genomic Epidemiology (Technical University of Denmark, Kgs. Lyngby, Denmark). *In silico* multi-locus sequence typing (MLST) by the Achtman’s MLST scheme database was performed with online resource MLST 2.0 of the Center for Genomic Epidemiology (Technical University of Denmark, Kgs. Lyngby, Denmark) [27]. The prophage regions in the chromosome were identified by online resource PHASTER (University of Alberta, Edmonton, AB, Canada) [38]. Web resource BLAST was used for homologous plasmids searching (National Center for Biotechnology Information, Bethesda, MD, USA) [39]. Online resource Genomics %G~C Content Calculator [40] was used to calculate GC content. Whole-genome alignments were performed using Mauve v. 2015-02-26 [41], Artemis Comparison Tool (Oxford University, Oxford, GB) [42], and BRIG v. 0.95 (https://brig.sourceforge.net/, access on 5 September 2022) [43]. Phylogenetic trees were obtained with core SNPs identified by WOMBAC (https://github.com/tseemann/wombac, access on 5 September 2022) [44] and were visualized with SplitsTree4 (https://github.com/husonlab/splitstree4, access on 5 September 2022) [45] and FigTree v. 1.4.4 (http://tree.bio.ed.ac.uk/software/figtree/, access on 5 September 2022) [46] using NJ method. Snippy software (https://github.com/tseemann/snippy, access on 5 September 2022) [47] was used to obtain variant calling into assembled chromosomes and plasmids with default parameters. The *stx2a* prophage sequences were extracted from the genomes using PHASTER (University of Alberta, Edmonton, AB, Canada) with manual detection of *att* sites by BLAST. The R package [48] with ggplot2 library (https://www.r-project.org/, access on 5 September 2022) [49] was used to calculate and visualize the distribution of SNPs, insertions, deletions, and complex in *E. coli* chromosome.

Whole-genome sequence of *E. coli* strain SCPM-O-B-9427 was submitted into GenBank database (National Center for Biotechnology Information, Bethesda, MD, USA): chromosome (CP086259) and five plasmids: pB-9427-1 (CP086260), pB-9427-2 (CP086261), pB-9427-3 (CP086262), pB-9427-4 (CP086263), and pB-9427-5 (CP086264).

Complete genomes of EAHEC strains *Escherichia coli* O104:H4 str. 2011C-3493: chromosome (CP003289), plasmid pAA-EA11 (CP003291), plasmid pESBL-EA11 (CP003290), plasmid pG-EA11 (CP003292), and *Escherichia coli* O104:H4 str. 2009EL-2050: chromosome (CP003297), plasmid pAA-09EL50 (CP003299), plasmid p09EL50 (CP003298), plasmid pG-09EL50 (CP003300) were used for comparative genomic analysis.

## 5. Conclusions

This study revealed the genetic properties of the new hybrid enteroaggregative/Shiga-toxin-producing (EAHEC) strain *E. coli* of O181:H4 (SCPM-O-B-9427) obtained from the patient with HUS in St. Petersburg (Russian Federation) in July 2018. The complete genome assembly of the strain SCPM-O-B-9427 contains one chromosome (5,268,110 bp) and five plasmids (pB-9427-1 83,340 bp; pB-9427-2 75,544 bp; pB-9427-3 51,013 bp; pB-9427-4 7939 bp; pB-9427-5 6728 bp). On the phylogenetic tree, the strain SCPM-O-B-9427 forms a close group with the strains *E. coli* O181:H4 and *E. coli* O104:H4. The comparison of the strain SCPM-O-B-9427 with *E. coli* O104:H4 strains 2011C-3493 (which caused the large German outbreak in 2011) and 2009EL-2050 (which was isolated in the Republic of Georgia in 2009) showed that all of them have the almost identical sets of virulence genes. The chromosomal architectures of the hybrid strains SCPM-O-B-9427, 2009EL-2050, and 2011C-3493 were very similar in overall structure despite one extended inverted region in the strain SCPM-O-B-9427. However, each of these strains was distinct, with extended unique regions in the genome. The comparative analysis of *stx2a* prophages, an important virulence determinant, showed that prophage of the strain SCPM-O-B-9427 is highly homologous to the prophage of the strain 2009EL-2050 and distinguished from the prophage of the strain 2011C-3493. The analysis of the plasmid pAA homologous revealed that the plasmids have no rearrangements; the size of the plasmid pB-9427-2 was 1331 bp bigger due to an additional insertion caused by IS4-like element IS421 family transposase.

Notably, the strains SCPM-O-B-9427 and 2009EL-2050 did not cause enormous outbreaks compared to the strain 2011C-3493. Therefore, hybrid strains producing Stx2 and the aggregative adherence-mediating fimbriae simultaneously could have a potential threat to humans. Combination of these virulence factors increase the pathogenic potential due to the damaging effects on the intestinal epithelium and colonization of the gastrointestinal tract. Therefore, it is necessary to undertake investigations of new genetic variants of pathogenic *E. coli* to detect their spreading among people and in environments.

## Figures and Tables

**Table 1:** Antimicrobial susceptibility of EAHEC strain SCPM-O-B-9427.

Antimicrobials	MIC, mg/L	Interpretation
Ampicillin	16	R ^1^
Amoxicillin/clavulanic acid	4	S ^2^
Cefotaxime	≤1	S
Ceftazidime	≤1	S
Cefoperazone/sulbactam	≤8	S
Cefepime	≤1	S
Aztreonam	≤1	S
Imipenem	≤1	S
Meropenem	≤0.25	S
Amikacin	≤2	S
Gentamicin	≤1	S
Netilmicin	2	S
Ciprofloxacin	1	R
Fosfomycin	≤16	S
Nitrofurantoin	≤16	S
Trimethoprim/sulfamethoxazole	≤20	S

^1^ Resistant; ^2^ susceptible.

**Table 2:** Major genome characteristics of the EAHEC strains SCPM-O-B-9427, 2011C-3493, and 2009EL-2050.

Feature/Strain	SCPM-O-B-9427	2011C-3493	2009EL-2050
GenBank chromosome	CP086259	CP003289	CP003297
Region	Russian Federation	Germany	Georgia
Isolation year	2018	2011	2009
Serotype	O181:H4	O104:H4	O104:H4
ST	ST678	ST678	ST678
*stx* subtype	2a	2a	2a
*aggDCBA* cluster	+	+	+
Chromosome size, bp	5,268,110	5,273,097	5,253,138
GC content of chromosome, %	50.7	50.7	50.7
Genes (total)	5408	5314	5325
CDS (total)	5283	5191	5197
Genes (RNA)	125	123	128
rRNAs	8, 7, 7 (5S, 16S, 23S)	8, 7, 7 (5S, 16S, 23S)	8, 7, 7 (5S, 16S, 23S)
tRNAs	95	92	97
Pseudogenes (total)	297	227	237
Plasmids	-	-	p09EL50 (109,274 bp)
	-	pESBL-EA11 (88,544 bp)	-
	pB-9427-1 (83,340 bp)	-	-
	pB-9427-2 (75,544 bp)	pAA-EA11 (74,213 bp)	pAA-09EL50 (74,213 bp)
	pB-9427-3 (51,013 bp)	-	
	pB-9427-4 (7939 bp)	-	
	pB-9427-5 (6728 bp)	-	
	-	pG-EA11 (1549 bp)	pG-09EL50 (1549 bp)

Note: ST, sequence type; CDS, coding sequence.

**Table 3:** Feature of unique regions of EAHEC strains SCPM-O-B-9427, 2009EL-2050, and 2011C-3493 detected using Artemis.

Region	Sequence, Kbp/GC Content. %			Note
	SCPM-O-B-9427	2009EL-2050	2011C-3493	
R1	52.0/47.5	45.0/56.5	44.5/56.8	Part of R1 are incomplete prophages and in the strain SCPM-O-B-9427 are related to *Enterobacteria phage* BP-4795 (NC_004813) and in other strains to *Enterobacteria phage* P1 (NC_005856).
R2	11.7/31.4	9.6/30.7	9.6/30.7	The region contains the genes involved in lipopolysaccharide synthesis of O104 (the strains 2011C-3493 and 2009EL-2050) and O181 (the strain SCPM-O-B-9427) antigen gene clusters.
R3	33.9/52.6	33.9/52.6	32.6/53.0	The region of the strain 2011C-3493 is related to *Bacteriophage* sp. isolate ctdA53 (BK038108) and the regions of the strains SCPM-O-B-9427 and 2009EL-2050 to *Escherichia phage* Lambda_ev017 (NC_049948).
R4	21.6/51.4	20.1/52.3	20.1/52.3	The region contains the chromosomal genes, IS elements, and genes coding the hypothetical proteins.
R5	61.6/50.2	60.6/50.2	60.9/50.2	The region is the *stx2a* prophage.
R6	65.6/49.2	-	-	The region contains the chromosome genes, part of them coding the type II secretion system.
R7	-	11.0/43.3	27.8/45.7	The region contains the genes related to *Enterobacteria phage* Sf6 (NC_005344).
R8	-	44.6/50.5	44.6/50.5	The region contains the genes related to intact *Enterobacteria phage* lambda (NC_001416).
R9	-	14.8/53.1	30.2/49.0	The region coding the chromosome genes that are partly missed in the strain 2009EL-2050.

**Table 4:** Plasmids of the strain SCPM-O-B-9427.

Plasmid	Accession Number	GC Contain (%)	Homolog	Query Cover (%)	Percentage of Identity (%)	Note
pB-9427-1	CP086260	53.3	*Escherichia coli* FDAARGOS_1300 plasmid unnamed3 (CP069999)—82,270 bp	98	99.53	F plasmid
pB-9427-2	CP086261	47.2	*Escherichia coli* O104:H4 FDAARGOS_348 plasmid unnamed2 (CP022087)—75,559 bp	100	99.91	pAA plasmid
pB-9427-3	CP086262	49.6	*Escherichia coli* SJ7 plasmid pSJ7-2 (CP051658)—53,543 bp	94	99.23	Contains genes encoding the type IV conjugative transfer system
pB-9427-4	CP086263	41.6	*Escherichia coli* RHBSTW-00895 plasmid pRHBSTW-00895_4 (CP056266)—7939 bp	100	99.67	Contains genes related to mobilization proteins
pB-9427-5	CP086264	51.3	*Shigella sonnei* 500867 plasmid pSSE2 (KP979588)—6728 bp	100	100	Contains plasmid-borne E-type colicin

## Data Availability

Whole-genome sequence of *E. coli* strain SCPM-O-B-9427 was submitted into GenBank database: chromosome (CP086259) and five plasmids: pB-9427-1 (CP086260), pB-9427-2 (CP086261), pB-9427-3 (CP086262), pB-9427-4 (CP086263), and pB-9427-5 (CP086264).

## References

[1] Eckburg P.B., Bik E.M., Bernstein C.N., Purdom E., Dethlefsen L., Sargent M., Gill S.R., Nelson K.E., Relman D.A. (2005). Diversity of the human intestinal microbial flora. Science.

[2] Stromberg Z.R., Johnson J.R., Fairbrother J.M., Kilbourne J., Van Goor A., Curtiss R., Mellata M. (2017). Evaluation of *Escherichia coli* isolates from healthy chickens to determine their potential risk to poultry and human health. PLoS ONE.

[3] Köhler C.D., Dobrindt U. (2011). What defines extraintestinal pathogenic *Escherichia coli*?. Int. J. Med. Microbiol..

[4] Clements A., Young J.C., Constantinou N., Frankel G. (2012). Infection strategies of enteric pathogenic *Escherichia coli*. Gut Microbes.

[5] Nyong E.C., Zaia S.R., Allué-Guardia A., Rodriguez A.L., Irion-Byrd Z., Koenig S., Feng P., Bono J.L., Eppinger M. (2020). Pathogenomes of Atypical Non-shigatoxigenic *Escherichia coli* NSF/SF O157:H7/NM: Comprehensive Phylogenomic Analysis Using Closed Genomes. Front. Microbiol..

[6] Chaudhuri R.R., Henderson I.R. (2012). The evolution of the *Escherichia coli* phylogeny. Infect. Genet. Evol..

[7] Dixit P.D., Pang T.Y., Studier F.W., Maslov S. (2015). Recombinant transfer in the basic genome of *Escherichia coli*. Proc. Natl. Acad. Sci. USA.

[8] Desvaux M., Dalmasso G., Beyrouthy R., Barnich N., Delmas J., Bonnet R. (2020). Pathogenicity Factors of Genomic Islands in Intestinal and Extraintestinal *Escherichia coli*. Front. Microbiol..

[9] Manges A.R., Geum H.M., Guo A., Edens T.J., Fibke C.D., Pitout J. (2019). Global Extraintestinal Pathogenic *Escherichia coli* (ExPEC) Lineages. Clin. Microbiol. Rev..

[10] Pakbin B., Brück W.M., Rossen J. (2021). Virulence Factors of Enteric Pathogenic *Escherichia coli*: A Review. Int. J. Mol. Sci..

[11] Jesser K.J., Levy K. (2020). Updates on defining and detecting diarrheagenic *Escherichia coli* pathotypes. Curr. Opin. Infect. Dis..

[12] Bolukaoto J.Y., Singh A., Alfinete N., Barnard T.G. (2021). Occurrence of Hybrid Diarrhoeagenic *Escherichia coli* Associated with Multidrug Resistance in Environmental Water, Johannesburg, South Africa. Microorganisms.

[13] Díaz-Jiménez D., García-Meniño I., Herrera A., García V., López-Beceiro A.M., Alonso M.P., Blanco J., Mora A. (2020). Genomic Characterization of *Escherichia coli* Isolates Belonging to a New Hybrid aEPEC/ExPEC Pathotype O153:H10-A-ST10 eae-beta1 Occurred in Meat, Poultry, Wildlife and Human Diarrheagenic Samples. Antibiotics.

[14] Valiatti T.B., Santos F.F., Santos A., Nascimento J., Silva R.M., Carvalho E., Sinigaglia R., Gomes T. (2020). Genetic and Virulence Characteristics of a Hybrid Atypical Enteropathogenic and Uropathogenic *Escherichia coli* (aEPEC/UPEC) Strain. Front. Cell. Infect. Microbiol..

[15] Santos A., Santos F.F., Silva R.M., Gomes T. (2020). Diversity of Hybrid- and Hetero-Pathogenic *Escherichia coli* and Their Potential Implication in More Severe Diseases. Front. Cell. Infect. Microbiol..

[16] Rasko D.A., Webster D.R., Sahl J.W., Bashir A., Boisen N., Scheutz F., Paxinos E.E., Sebra R., Chin C.S., Iliopoulos D. (2011). Origins of the *E. coli* strain causing an outbreak of hemolytic-uremic syndrome in Germany. N. Engl. J. Med..

[17] Tietze E., Dabrowski P.W., Prager R., Radonic A., Fruth A., Aura P., Nitsche A., Mielke M., Flieger A. (2015). Comparative genomic analysis of two novel sporadic Shiga toxin-producing *Escherichia coli* O104:H4 strains isolated 2011 in Germany. PLoS ONE.

[18] Wald M., Rieck T., Nachtnebel M., Greute’laers B., an der Heiden M., Altmann D., Hellenbrand W., Faber M., Frank C., Schweickert B. (2011). Enhanced surveillance during a large outbreak of bloody diarrhoea and haemolytic uraemic syndrome caused by Shiga toxin/verotoxin-producing *Escherichia coli* in Germany, May to June 2011. Euro Surveill..

[19] Chokoshvili O., Lomashvili K., Malakmadze N., Geleishvil M., Brant J., Imnadze P., Chitadze N., Tevzadze L., Chanturia G., Tevdoradze T. (2014). Investigation of an outbreak of bloody diarrhea complicated with hemolytic uremic syndrome. J. Epidemiol. Glob. Health.

[20] Ahmed S.A., Awosika J., Baldwin C., Bishop-Lilly K.A., Biswas B., Broomall S., Chain P.S., Chertkov O., Chokoshvili O., Coyne S. (2012). Threat Characterization Consortium. Genomic comparison of *Escherichia coli* O104:H4 isolates from 2009 and 2011 reveals plasmid, and prophage heterogeneity, including shiga toxin encoding phage *stx2*. PLoS ONE.

[21] Nowrouzian F.L., Wold A.E., Adlerberth I. (2005). *Escherichia coli* strains belonging to phylogenetic group B2 have superior capacity to persist in the intestinal microflora of infants. J. Infect. Dis..

[22] Scheutz F., Cheasty T., Woodward D., Smith H.R. (2004). Designation of O174 and O175 to temporary O groups OX3 and OX7, and six new *E. coli* O groups that include Verocytotoxin-producing *E. coli* (VTEC): O176, O177, O178, O179, O180 and O181. APMIS.

[23] Ballem A., Gonçalves S., Garcia-Meniño I., Flament-Simon S.C., Blanco J.E., Fernandes C., Saavedra M.J., Pinto C., Oliveira H., Blanco J. (2020). Prevalence and serotypes of Shiga toxin-producing *Escherichia coli* (STEC) in dairy cattle from Northern Portugal. PLoS ONE.

[24] Eklund M., Scheutz F., Siitonen A. (2001). Clinical isolates of non-O157 Shiga toxin-producing *Escherichia coli*: Serotypes, virulence characteristics, and molecular profiles of strains of the same serotype. J. Clin. Microbiol..

[25] García-Aljaro C., Muniesa M., Blanco J.E., Blanco M., Blanco J., Jofre J., Blanch A.R. (2005). Characterization of Shiga toxin-producing *Escherichia coli* isolated from aquatic environments. FEMS Microbiol. Lett..

[26] Ori E.L., Takagi E.H., Andrade T.S., Miguel B.T., Cergole-Novella M.C., Guth B., Hernandes R.T., Dias R., Pinheiro S., Camargo C.H. (2018). Diarrhoeagenic *Escherichia coli* and *Escherichia albertii* in Brazil: Pathotypes and serotypes over a 6-year period of surveillance. Epidemiol. Infect..

[27] Wirth T., Falush D., Lan R., Colles F., Mensa P., Wieler L.H., Karch H., Reeves P.R., Maiden M.C., Ochman H. (2006). Sex and virulence in *Escherichia coli*: An evolutionary perspective. Mol. Microbiol..

[28] Berger P., Knödler M., Förstner K.U., Berger M., Bertling C., Sharma C.M., Vogel J., Karch H., Dobrindt U., Mellmann A. (2016). The primary transcriptome of the Escherichia coli O104:H4 pAA plasmid and novel insights into its virulence gene expression and regulation. Sci. Rep..

[29] Navarro-Garcia F. (2014). *Escherichia coli* O104:H4 pathogenesis: An enteroaggregative *E. coli*/Shiga toxin-Producing *E. coli* explosive cocktail of high virulence. Microbiol. Spectr..

[30] Beghain J., Bridier-Nahmias A., Le Nagard H., Denamur E., Clermont O. (2018). ClermonTyping: An easy-to-use and accurate in silico method for Escherichia genus strain phylotyping. Microb Genom..

[31] Wilson K. (2001). Preparation of genomic DNA from bacteria. Curr. Protoc. Mol. Biol..

[32] Oxford Nanopore Technologies. https://nanoporetech.com/.

[33] Wick R.R., Judd L.M., Gorrie C.L., Holt K.E. (2017). Unicycler: Resolving bacterial genome assemblies from short and long sequencing reads. PLoS Comput. Biol..

[34] Tatusova T., DiCuccio M., Badretdin A., Chetvernin V., Nawrocki E.P., Zaslavsky L., Lomsadze A., Pruitt K.D., Borodovsky M., Ostell J. (2016). NCBI prokaryotic genome annotation pipeline. Nucleic. Acids. Res..

[35] Joensen K.G., Tetzschner A.M., Iguchi A., Aarestrup F.M., Scheutz F. (2015). Rapid and easy in silico serotyping of *Escherichia coli* using whole genome sequencing (WGS) data. J. Clin. Microbiol..

[36] Malberg Tetzschner A.M., Johnson J.R., Johnston B.D., Lund O., Scheutz F. (2020). In Silico Genotyping of *Escherichia coli* Isolates for Extraintestinal Virulence Genes by Use of Whole-Genome Sequencing Data. J. Clin. Micobiol..

[37] Bortolaia V., Kaas R.S., Ruppe E., Roberts M.C., Schwarz S., Cattoir V., Philippon A., Allesoe R.L., Rebelo A.R., Florensa A.F. (2020). ResFinder 4.0 for predictions of phenotypes from genotypes. J. Antimicrob. Chemother..

[38] Arndt D., Grant J.R., Marcu A., Sajed T., Pon A., Liang Y., Wishart D.S. (2016). PHASTER: A better, faster version of the PHAST phage search tool. Nucleic. Acids Res..

[39] Camacho C., Coulouris G., Avagyan V., Ma N., Papadopoulos J., Kevin Bealer K., Madden T.L. (2009). BLAST+: Architecture and applications. BMC Bioinform..

[40] Genomics %G~C Content Calculator—Science Buddies. https://www.sciencebuddies.org/science-fair-projects/references/genomics-g-c-content-calculator.

[41] Darling A.E., Mau B., Perna N.T. (2010). progressiveMauve: Multiple genome alignment with gene gain, loss and rearrangement. PLoS ONE.

[42] Carver T.J., Rutherford K.M., Berriman M., Rajandream M.A., Barrell B.G., Parkhill J. (2005). ACT: The Artemis Comparison Tool. Bioinformatics.

[43] Alikhan N.F., Petty N.K., Ben Zakour N.L., Beatson S.A. (2011). BLAST Ring Image Generator (BRIG): Simple prokaryote genome comparisons. BMC Genom..

[44] Wombac. https://github.com/tseemann/wombac.

[45] Huson D.H., Bryant D. (2006). Application of Phylogenetic Networks in Evolutionary Studies. Mol. Biol. Evol..

[46] Molecular Evolution, Phylogenetics and Epidemiology. http://tree.bio.ed.ac.uk/software/figtree/.

[47] Snippy. https://github.com/tseemann/snippy.

[48] R Core Team (2018). R: A Language and Environment for Statistical Computing.

[49] Wickham H. (2016). Ggplot2: Elegant Graphics for Data Analysis.

